# Genome-wide association analysis of rheumatoid arthritis data via haplotype sharing

**DOI:** 10.1186/1753-6561-3-s7-s30

**Published:** 2009-12-15

**Authors:** Andrew S Allen, Glen A Satten

**Affiliations:** 1 Department of Biostatistics and Bioinformatics and Duke Clinical Research Institute, Duke University, DUMC 3850, Durham, North Carolina 27710 USA; 2 National Center for Chronic Disease Prevention and Health Promotion, Centers for Disease Control and Prevention, 1600 Clifton Road, Atlanta, Georgia 30333 USA

## Abstract

We present computationally simple association tests based on haplotype sharing that can be easily applied to genome-wide association studies, while allowing use of fast (but not likelihood-based) haplotyping algorithms, and properly accounting for the uncertainty introduced by using inferred haplotypes. We also give haplotype sharing analyses that adjust for population stratification. We apply our methods to a genome-wide association study of rheumatoid arthritis available as Problem 1 of Genetic Analysis Workshop 16. In addition to the HLA region on chromosome 6, we find genome-wide significant signals at 7q33 and 13q31.3. These regions contain genes with interesting potential connections with rheumatoid arthritis and are not identified using single single-nucleotide polymorphism methods.

## Background

The large number of markers tested in a genome-wide association study (GWAS) has forced a simplification of analytic approaches. While sophisticated methodology may be used to adjust for multiple comparisons and population stratification, the sheer number of tests in a GWAS requires that each test be fairly simple; currently, most studies are analyzed by computing a simple test such as the Cochran-Armitage trend test at each locus. Methods that account for the special features of genetic association studies, yet remain computationally feasible for genome-wide analysis, are desirable because they may lead to increased power to detect associations. Haplotype sharing is a simple concept that attempts to translate between population genetics and genetic epidemiology. In brief, for recent mutations that cause disease, we would expect that haplotypes of case participants would be more similar to each other in the immediate region of a mutation than they would be to the haplotypes of control participants, suggesting a comparison of sharing in a region between cases and controls. We have recently proposed a class of computationally simple association tests based on haplotype sharing that can be easily applied to case-control studies on the genome-wide scale. The computational simplicity allows for quick assessment of genome-wide significance while adjusting for population stratification via a stratified analysis employing the two-step method of Epstein et al. [1]. We apply this methodology to the rheumatoid arthritis (RA) whole-genome association data available as Problem 1 of Genetic Analysis Workshop 16.

## Methods

We begin by giving an overview of the class of test statistics we consider. A more detailed presentation of our approach can be found in Allen and Satten [2]. Let *d*_*i *_and *z*_*i *_indicate disease status and stratum membership, respectively, for the *i*^th ^individual. We consider haplotypes of fixed length *L *so that there are  = 2^*L *^possible haplotypes to consider. Let , , and  be -dimensional vectors having *j*^th ^components given by the frequency of haplotypes, in stratum *z*, among the cases, controls, and the entire sample, respectively. Define the  ×  matrix  whose (*j*, *j*') element is the sharing between the *j*^th ^and *j*'^th ^haplotypes about a fixed locus *k*. Here we measure sharing by the maximum information length contrast [3] metric which counts the number of single-nucleotide polymorphisms (SNPs) that the *j*^th ^and *j*'^th ^haplotypes share identically by state in a window centered at locus *k*. To simplify notation we drop the index *k*, though it should be understood that all quantities are computed relative to a given locus *k*. For each locus, we consider statistics of the form

where γ is a -dimensional vector that defines the member of the class and *w*_*z *_is a scalar weight function. Implicit in these definitions is a "working" model φ (*h*|*g*) the probability of diplotype *h *given multilocus genotype *g*. This model is used when we compute , and , the distribution of haplotypes consistent with the *i*^th ^individual's observed genotype data, under phase ambiguity. It is not hard to show that (1) can be derived as the efficient score of a model within the class of models previously studied by Allen and Satten [4]. As a consequence, they remain valid even if the "working" model φ (*h*|*g*) is misspecified. Further, it is not necessary to adjust the variance of our test statistic to account for uncertainty in haplotype frequencies. We exploit these facts by choosing computationally fast, though perhaps inconsistent, estimates of φ (*h*|*g*) secure in the fact that such a choice will not affect the validity of our testing procedure. Here we consider two members of the class given by Eq. (1): first, the "*p*" statistic in which *γ*_*z *_= , and the "cross" statistic in which γ_*z *_= ().

We can interpret the "*p*" and "cross" statistics as testing for differences in sharing between cases and controls in the direction of  and , respectively. The "*p*" statistic has the simple variance estimator,

For the "cross" statistic the situation is a bit more complex. We can show that  is distributed as a mixture of independent χ^2 ^variates with weights given by the eigenvalues of . We approximate this distribution using the three-moment approximation of Imhoff [5], which has the computational advantage of only depending on the trace of ()^*m *^for *m *= 1, 2, 3.

We applied our proposed haplotype sharing methodology to the RA data provided in Genetic Analysis Workshop 16 Problem 1. This data set has been described elsewhere but, in brief, contains genotypes that include over 545,000 unique SNPs for 868 patients with RA and 1194 controls.

### Genotypes, haplotypes, and quality control

Following Fellay et al. [6], we excluded data from SNPs that had extensive missingness (missingness >10), deviations from Hardy-Weinberg equilibrium (*p*-value < 0.001 in controls), and low minor allele frequency (<0.2%). After this quality control (QC) filtering, 530,817 SNPs remained. Using the software package PLINK [7], we confirmed that all pairs of individuals shared less than 12.5% of SNP alleles (the threshold used by Fellay et al.) identically by descent. Thus, no individuals were excluded for cryptic relatedness. No individuals were excluded for missingness.

We used a computationally efficient estimator of the distribution of haplotypes given the observed genotype data φ (*h*|*g*). The phasing program ent [8] was used to impute a single diplotype for each chromosome of each study participant. For a given window, the empirical distribution of the imputed haplotypes composed of SNPs in the window was used as a simple haplotype frequency estimator. Haplotype frequency estimates computed in this way were then used in specifying the "working" model for φ (*h*|*g*), assuming Hardy-Weinberg equilibrium. We note that although we imputed individual haplotypes as a simple way to estimate φ (*h*|*g*), that when computing , , and , we summed individual contributions over φ (*h*|*g*), and therefore, explicitly accounted for phase ambiguity. As discussed above, misspecification of φ (*h*|*g*) will not affect the validity of the haplotype-sharing tests.

### Adjustment for confounding due to population stratification

We used the stratification score of Epstein et al. [1] to adjust our analyses for confounding due to population stratification. In Epstein et al. [1], partial least squares (PLS) were used to estimate the stratification score. Here we used a modified principal-component (PC) approach [6] in place of PLS. This modified PC approach captures the large-scale genetic variation in the data by minimizing the influence of a few high linkage disequilibrium (LD) regions from dominating the first few PCs. This is accomplished by excluding SNPs that reside in regions of known high LD from the PC analysis and then further pruning the PC SNP set to minimize the LD between the remaining SNPs [6]. Using the first few PCs, four individuals (D0009459, D0011466, D0012257, and D0012446) were found to be significant outliers, suggesting appreciable non-white ancestry. These individuals were excluded from subsequent analyses and when the PC analysis was repeated, no further outliers were identified. The first ten PCs were then used in a logistic model of disease to estimate each individual's stratification score--their predicted probability of being a case given the genomic information contained in the PCs. Four strata were then formed based on the quantiles of the stratification scores, for use in a stratified haplotype-sharing analysis. For each locus *k*, we used the sample size in the *z*^th ^stratum as the weight function *w*_*z *_in Eq. (1).

### Genome-wide haplotype sharing analysis

The final analysis data set consisted of 517,843 autosomal SNP genotypes that passed QC from 868 case participants with RA and 1190 control participants. To this data set we applied two stratified haplotype-sharing tests: the cross test and the *p *test. Each test was calculated using a sliding window of seven SNPs. We measured inflation of test statistics due to residual population stratification by the variance inflation factor (VIF), defined as ratio of the median of the observed and expected chi-square statistics across the genome. Permutation tests were conducted by randomly permuting case/control labels within each strata and then capturing the minimum *p*-value of each statistic across the genome for each permutation. We estimated genome-wide significance by comparing the observed *p*-values to this permutation distribution.

## Results

**Figure 1:**
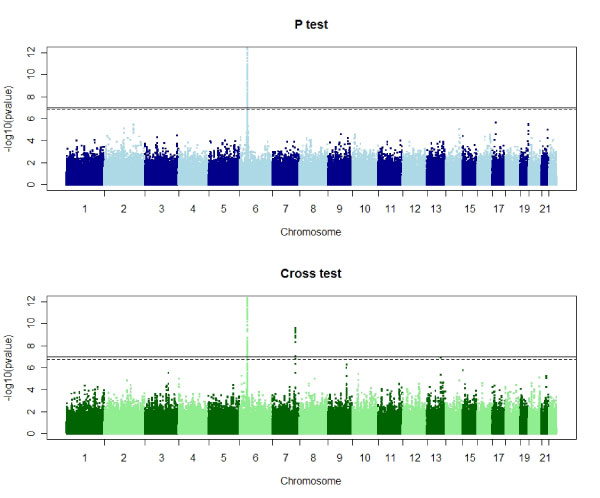
**Manhattan plot of -log_10_(*p*-value)s of haplotype sharing tests for autosomal SNPs passing QC filtering**. Solid horizontal line represents Bonferroni genome-wide threshold. Dashed horizontal line represents genome-wide threshold estimated via permutation.

We first confirmed the stratification score controlled for inflation due to population stratification. An unadjusted single locus analysis [9] showed a VIF of 1.44, suggesting that significant stratification exist in these data. The stratified *p *and cross tests had VIFs of 1.03 and 1.04, respectively, suggesting minimal residual inflation. The results of these stratified haplotype sharing analyses across autosomal SNPs are given in Figure 1.

Outside the HLA region on chromosome 6, the *p *test shows no further regions associated with RA. However, the cross test implicated two genomic regions having -log_10_(*p*-value)s that exceed the permutation-based genome-wide threshold. These regions are: 7q33 (windows centered at rs6467709, rs6964837, rs834092, rs834082, rs834067, rs1646366, rs834063, and rs864434), and 13q31.3 (window centered at rs9584093). Each of these regions contain genes with interesting potential connections with RA. The region on chromosome 7 is adjacent to the pleiotrophin gene (PTN), which has been found to be up-regulated in synovial tissues from patients with RA [10]. The region on chromosome 7 contains glypican 6 (GPC 6). Glypicans have been shown to be expressed differentially in chronically inflamed synovium [11].

## Conclusion

Apart from the HLA region on chromosome 6, none of the regions implicated in our analysis were found by a single-locus GWA analysis that was appropriately corrected for population stratification [9]. This suggests that haplotype-based methods should have a role in the analysis of GWAS. The current approach of single-locus tests, possibly followed by a small-scale application of haplotype methods in candidate regions or regions where the single-SNP results are significant or almost significant may miss regions where a haplotype-based approach would find a signal. More generally, the strategy of evaluating haplotype methods by evaluating their performance in regions implicated by single-SNP methods may result in the false impression that single-SNP methods out-perform haplotype-based methods.

## List of abbreviations used

GPC 6: Glypican 6; GWAS: Genome-wide association study; LD: Linkage disequilibrium; PC: Principal component; PLS: Partial least squares; QC: Quality control; RA: Rheumatoid arthritis; SNP: Single-nucleotide polymorphism; VIF: Variance inflation factor

## Competing interests

The authors declare that they have no competing interests.

## Authors' contributions

ASA and GAS conceived the study and planned the analyses. ASA analyzed the data. ASA and GAS wrote the manuscript.
